# Low dissolved oxygen supply functions as a global regulator of the growth and metabolism of *Aurantiochytrium* sp. PKU#Mn16 in the early stages of docosahexaenoic acid fermentation

**DOI:** 10.1186/s12934-023-02054-w

**Published:** 2023-03-15

**Authors:** Lu Liu, Xingyu Zhu, Huike Ye, Yingying Wen, Biswarup Sen, Guangyi Wang

**Affiliations:** 1 Center for Marine Environmental Ecology, School of Environmental Science and Engineering, Tianjin University, Tianjin, 300072 China; 2 Key Laboratory of Systems Bioengineering (Ministry of Education), Tianjin University, Tianjin, 300072 China; 3 Center for Biosafety Research and Strategy, Tianjin University, Tianjin, 300072 China

**Keywords:** Thraustochytrids, Transcriptomics, Metabolomics, Central carbon metabolism, Amino acid metabolism, Fatty acids

## Abstract

**Background:**

Thraustochytrids accumulate lipids with a high content of docosahexaenoic acid (DHA). Although their growth and DHA content are significantly affected by the dissolved oxygen (DO) supply, the role of DO on the transcriptional regulation of metabolism and accumulation of intracellular metabolites remains poorly understood. Here we investigate the effects of three different DO supply conditions (10%, 30%, and 50%) on the fed-batch culture of the *Aurantiochytrium* PKU#Mn16 strain to mainly reveal the differential gene expressions and metabolite profiles.

**Results:**

While the supply of 10% DO significantly reduced the rates of biomass and DHA production in the early stages of fermentation, it achieved the highest amounts of biomass (56.7 g/L) and DHA (6.0 g/L) on prolonged fermentation. The transcriptome analyses of the early stage (24 h) of fermentation revealed several genes involved in the central carbon, amino acid, and fatty acid metabolism, which were significantly downregulated at a 10% DO level. The comparative metabolomics results revealed the accumulation of several long-chain fatty acids, amino acids, and other metabolites, supporting the transcriptional regulation under the influence of a low oxygen supply condition. In addition, certain genes involved in antioxidative systems were downregulated under 10% DO level, suggesting a lesser generation of reactive oxygen species that lead to oxidative damage and fatty acid oxidation.

**Conclusions:**

The findings of this study suggest that despite the slow growth and metabolism in the early stage of fermentation of *Aurantiochytrium* sp. PKU#Mn16, a constant supply of low dissolved oxygen can yield biomass and DHA content better than that with high oxygen supply conditions. The critical information gained in this study will help to further improve DHA production through bioprocess engineering strategies.

**Supplementary Information:**

The online version contains supplementary material available at 10.1186/s12934-023-02054-w.

## Background

Docosahexaenoic acid (DHA, C22:6) is an omega-3 long-chain polyunsaturated fatty acid (LC-PUFA), which has been shown to provide diverse human health benefits and is a well-recognized nutraceutical [1]. To meet the increasing demand for DHA and overcome the negative impacts of traditional resources, it is essential to establish a sustainable way for DHA production [2]. Like many other oleaginous microorganisms [3, 4], thraustochytrids—a group of unicellular marine heterotrophic protists—have attracted biotechnological interests because of their potential to accumulate high levels of DHA [1, 5, 6]. Interestingly, thraustochytrids have become one of the most potential microbial sources for DHA production at the industrial scale [6, 7]. However, the industrial production of DHA requires the identification and application of optimal bioprocess conditions to achieve the highest output [1, 8].

Dissolved oxygen (DO) is an important environmental condition influencing the growth and production of thraustochytrids [9–11]. Previous studies on thraustochytrids reported that a medium level of oxygen supply can yield the highest DHA productivity [12, 13]. For instance, an aeration rate of 1.0 vvm to *Schizochytrium* sp. HX-308 culture yielded the highest DHA productivity (206.7 mgL^−1^ h^−1^) compared with 0.5 vvm and 1.5 vvm [12]. With medium agitation speed (450 rpm) the *Schizochytrium* sp. 31 culture showed the highest concentration (21.26 g/L) of DHA, compared to that with 300 or 600 rpm [13]. Conversely, the highest volumetric oxygen transfer coefficient (k_L_a) of 1802 ± 105 h^−1^ achieved a DHA concentration of 28.93 g/L and productivity of 301 mgL^−1^ h^−1^, which were the best among those achieved with lower K_L_a values [14]. Furthermore, with an intermittent oxygen feeding method to maintain a 50% DO level for *Aurantiochytrium limacinum* SR21, a DHA concentration of 20.3 g/L was achieved [15]. However, these previous studies used different methodologies to control the oxygen levels, therefore it is somewhat difficult to compare their outputs and the effects of DO on DHA fermentation. In addition, most of the earlier investigations focused on the effects of various fermentation strategies for controlling the DO levels to maximize DHA production [10, 11, 15–18] and minimize the oxidative stress [9, 19, 20]. To determine more intuitively the effects of oxygen on the growth and metabolism of thraustochytrids, it is imperative to understand the dynamics of biomass and lipid accumulation under different DO levels. Besides, the transcriptional and metabolic changes within thraustochytrid cells in response to different DO levels remain poorly understood.

Two distinct pathways, the fatty acid synthase (FAS) pathway and PUFA synthase (PKS), are known to involve in the de novo biosynthesis of DHA in thraustochytrids [21, 22]. The former is an aerobic pathway and carries out a series of desaturation and elongation steps to introduce double bonds and extend the acyl chain. The other is an anaerobic pathway that synthesizes DHA from malonyl-CoA through the PKS-like machinery. Whether and how the DO levels affect the expression of biosynthetic genes involved in these two pathways, particularly, the ‘anaerobic’ pathway remain largely unknown. Comparative transcriptomics analysis of *Schizochytrium* sp. HX-308 culture between agitation of 300 rpm and 400 rpm suggested upregulation of genes associated with central carbon and fatty acid metabolism under 400 rpm agitation [23]. Particularly, a higher oxygen supply resulted in more NADPH and acetyl‑CoA production for cell growth and lipid synthesis. Furthermore, comparative metabolomics analysis of *Schizochytrium* sp. HX-308 culture indicated that a low oxygen level can yield significantly higher levels of succinate and several amino acids, suggesting their important roles in resisting oxygen deficiency and regulating DHA synthesis [24]. Interestingly, enhanced cell growth and PUFA production has been found to closely associate with the amino acid levels in engineered *Schizochytrium* sp. HX-RS [25]. Overall, these previous studies focused on the responses of thraustochytrids either at the transcriptional or metabolic level and did not provide a holistic view of cellular metabolism under the influence of different DO supply conditions.

In this study, the growth and DHA production of a previously isolated thraustochytrid strain (*Aurantiochytrium* sp. PKU#Mn16) were investigated in variable volume fed-batch fermentation at three different DO levels. The cellular responses to the different DO levels were further evaluated using comparative transcriptomics and metabolomics approaches. This study aimed to provide a comprehensive report on the impact of DO levels on growth, DHA production, and relevant metabolic pathways.

## Results

### Growth and lipid production under different DO levels

**Fig. 1:**
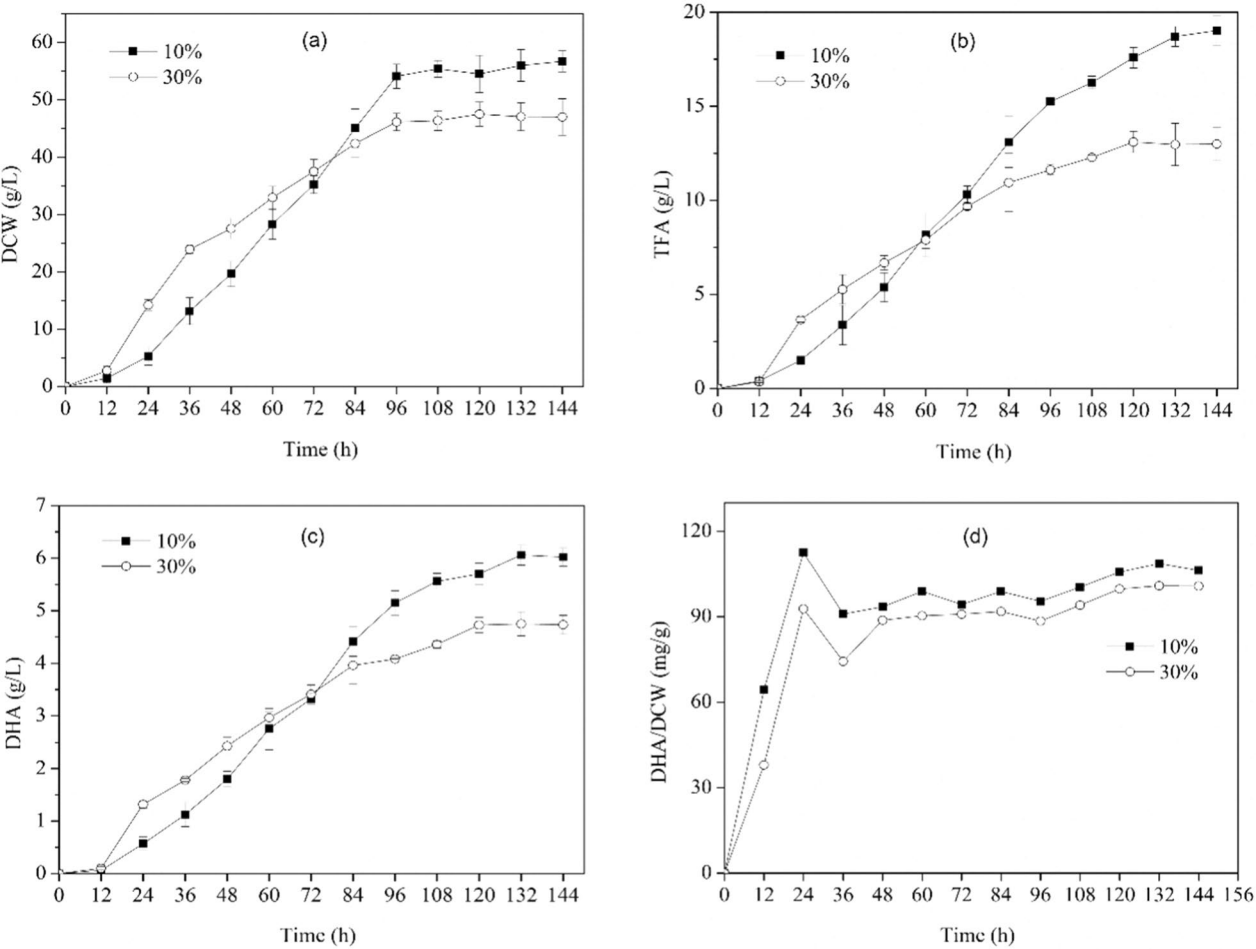
Dynamics of **a** biomass, **b** TFA, **c** DHA, and **d** DHA yield during fed-batch cultivation of *Aurantiochytrium* sp. PKU#Mn16 at 10% and 30% DO levels

To study the effects of DO on growth, TFA, and DHA production, variable-volume fed-batch fermentation experiments were conducted under three different DO levels (10%, 30%, and 50%). The specific growth rates (µ) of PKU#Mn16 culture cultivated under 10%, 30%, and 50% DO were 0.103 h^−1^, 0.111 h^−1^, and 0.116 h^−1^, respectively. At 10% oxygen saturation, the culture exhibited a comparatively delayed initiation of the exponential growth phase than those cultivated at 30% and 50% DO levels. Before the first feeding at 36 h, the biomass reached 13.2 ± 2.4 g/L, 23.9 ± 0.76 g/L, and 26.6 ± 0.34 g/L, respectively (Additional file 1: Table S1). These results indicated that 30% and 50% saturation levels can significantly increase biomass production in the early exponential phase. Furthermore, the cultures at 30% and 50% DO levels were fed twice, at 36 h and 48 h, whereas the culture under 10% level was fed once, at 48 h, during the exponential phase (Additional file 1: Fig. S1). This suggests that PKU#Mn16 culture under 30% and 50% DO levels consumed more amount of the carbon source than that under 10% DO level during exponential growth, resulting in higher biomass production. However, the culture under 50% DO level consumed a significantly lower amount of the carbon source during the 48 h–60 h period and thereafter reached the stationary growth phase (Additional file 1: Fig. S1). Interestingly, the cultures under 10% and 30% saturation reached their stationary phases much later at 96 h and meanwhile consumed more amounts of the carbon source (Fig. 1a). However, in the stationary phase, the biomass of PKU#Mn16 culture was significantly higher at 10% DO level than that at 30% and 50% DO levels. Overall, the biomass at 10%, 30%, and 50% DO levels ranged between 0 g/L–56.7 g/L, 0 g/L–47.5 g/L, and 0 g/L–38.5 g/L, respectively, during the fermentation period of 144 h (Additional file 1: Table S1). These findings suggest that although a 10% DO level can produce a higher amount of biomass at the end of fermentation, it significantly retards the growth in the exponential phase.

The TFA productivity by PKU#Mn16 culture at 10% DO level exhibited a much slower rate (0.092 g/L.h^−1^) at 24 h of fermentation than those cultivated at 30% (0.275 g/L.h^−1^) and 50% (0.253 g/L.h^−1^) DO level (Additional file 1: Fig. S2). Before the first feeding at 36 h, the TFA productions at 10%, 30%, and 50% DO levels were 3.37 ± 1.05 g/L, 5.27 ± 0.78 g/L, and 7.62 ± 0.56 g/L, respectively (Additional file 1: Table S1). However, after the first feeding, the production rate of culture at 10% DO level increased gradually and remained greater than those at 30% and 50% (Additional file 1: Fig. S2). The highest rates under 10%, 30%, and 50% DO levels were observed at 60 h (0.232 g/L.h^−1^), 24 h (0.275 g/L.h^−1^), and 36 h (0.352 g/L.h^−1^), respectively. The maximum TFA concentrations were obtained at 144 h (19.0 ± 0.77 g/L), 120 h (13.1 ± 0.56 g/L), and 120 h (15.2 ± 0.66 g/L), respectively (Additional file 1: Table S1, Fig. 1b). However, the best TFA yields (mg/g-DCW) at 10%, 30%, and 50% saturation were achieved at 12 h (360 ± 80 mg/g-DCW), 24 h (260 ± 10 mg/g-DCW), and 72 h (380 ± 30 mg/g-DCW), respectively.

**Fig. 2:**
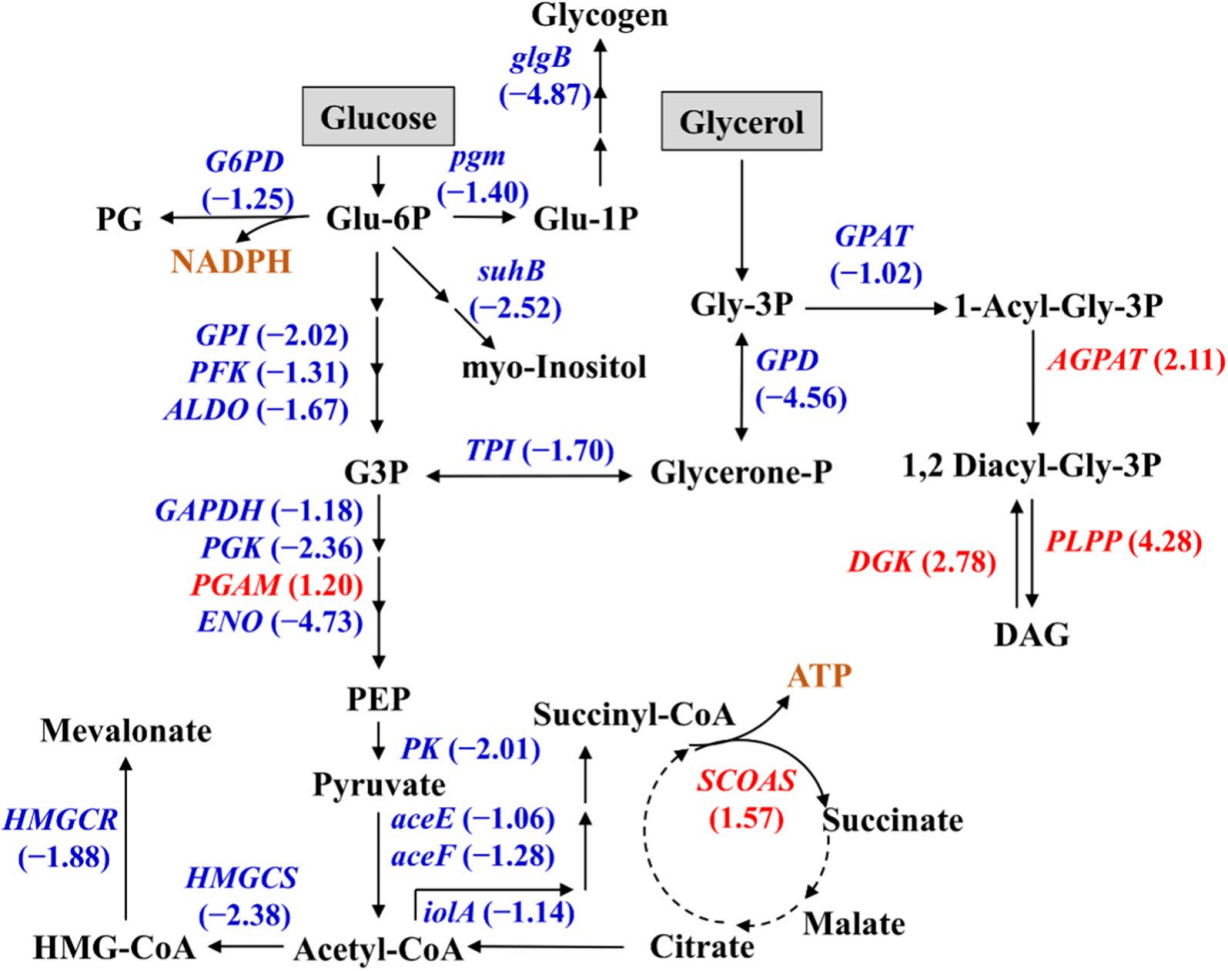
Differential gene expressions in central carbon metabolism between 10 and 30% DO levels at 24 h of fermentation. Genes in red or blue are supported by at least one transcript with significant regulation (|log_2_FC|≥ 1 and FDR < 0.05). Red: upregulated; Blue: downregulated. *pgm* phosphoglucomutase, *glgB* 1,4-alpha-glucan branching enzyme, *GPI* glucose-6-phosphate isomerase, *PFK* 6-phosphofructokinase, *ALDO* fructose-bisphosphate aldolase, *GAPDH* glyceraldehyde 3-phosphate dehydrogenase, *PGK* phosphoglycerate kinase, *PGAM* 2,3-bisphosphoglycerate-dependent phosphoglycerate mutase, *ENO* enolase, *PK* pyruvate kinase, *aceE* pyruvate dehydrogenase E1 component, *aceF* pyruvate dehydrogenase E2 component, *G6PD* glucose-6-phosphate dehydrogenase, *GPD* glycerol-3-phosphate dehydrogenase (NAD +), *SCOAS* succinyl-CoA synthetase, *TPI* triosephosphate isomerase, *HMGCS* hydroxymethylglutaryl-CoA synthase, *HMGCR* hydroxymethylglutaryl-CoA reductase (NADPH), *GPAT* glycerol-3-phosphate O-acyltransferase, *PLPP* phosphatidate phosphatase, *suhB* myo-inositol monophosphatase, *iolA* methylmalonate-semialdehyde dehydrogenase, *AGPAT* 1-acyl-sn-glycerol-3-phosphate acyltransferase, *DGK* diacylglycerol kinase. *Glu-6P* Glucose 6-phosphate, *PG* 6-Phospho-D-glucono-1,5-lactone, *G3P* Glyceraldehyde 3-phosphate, *PEP* Phosphoenolpyruvate, *Glycerol-3P* Glycerol 3-phosphate, *Glycerone*-P Glycerone phosphate, *HMG-CoA* Hydroxymethylglutaroyl coenzyme A, *DAG* 1,2 Diacylglycerol

As the major PUFA produced by PKU#Mn16 (Additional file 1: Fig. S3), DHA occupied more than 30% of TFA produced under 10%, 30%, and 50% DO levels during the exponential and stationary phases. The maximum DHA concentrations were 6.0 ± 0.19 g/L (132 h), 4.7 ± 0.23 (120 h), and 5.3 ± 0.23 g/L (108 h), respectively (Additional file 1: Table S1, Fig. 1c). The DHA yields at 10% (112.5 ± 14.08 mg/g-DCW) and 30% (92.8 ± 4.20 mg/g-DCW) DO levels were the highest at 24 h and were significantly different (Fig. 2d, Additional file 1: Table S1). Whereas at 50% DO level, the highest DHA yield was at 72 h (135.3 ± 1.40 mg/g-DCW). After the first feeding at 36 h, the DHA yields at 10% and 30% DO levels did not change drastically (Fig. 1d). These findings indicate that prolonged fermentation at low DO levels can produce more amount of DHA. However, a low DO level can significantly reduce DHA concentrations in the early stages of fermentation.

Overall, these findings suggest that a low DO level can markedly influence the rates of biomass and DHA production of PKU#Mn16 strain in the early stages of fermentation. Interestingly, at 10% DO level, although a contrastingly slower growth dynamics was evident, the biomass and DHA concentrations at the end of fermentation were the highest compared to that achieved at higher DO levels.

### De Novo assembly and functional annotation of PKU#Mn16 transcriptome

To investigate the mechanism underlying the differential dynamics of DHA production under different DO levels, the PKU#Mn16 transcriptome was analyzed between cultures cultivated under 10% and 30% DO levels. Sequencing of cDNA libraries generated an average of 46,255,072 and 43,535,297 raw reads for 10% and 30% DO level samples, respectively. After filtering, an average of 44,774,899 and 41,958,984 clean reads, respectively, were obtained. A total of 34,723 transcripts and 29,907 unigenes were further generated after assembly (Additional file 1: Table S2). The sequence length of transcripts/unigenes varied from 201 to 13,157 bp (Additional file 1: Fig. S4). The N50 values of transcripts and unigenes were 1963 bp and 2,024 bp, respectively. Functional annotation of all the unigenes revealed that 28.35%, 20.02%, 29.18%, 25.73%, 32.95%, and 25.77% of them match with the known proteins in GO, KEGG, Pfam, SwissProt, eggNOG, and NR databases (Additional file 1: Table S2). Furthermore, a total of 7,053 unigenes were identified as DEGs, among which, 3,107 were upregulated and 3,946 were downregulated (Additional file 1: Table S2).

### Transcriptional regulation of central carbon and amino acid metabolism

The comparative transcriptomics results showed that certain genes involved in the central carbon metabolism, including glycolysis, glycogen metabolism, oxidative phosphorylation, and TCA cycle were differentially regulated between 10 and 30% DO levels (Fig. 2). The DEGs encoding glycolytic enzymes included glucose-6-phosphate isomerase, phosphofructokinase, aldolase, glyceraldehyde 3-phosphate dehydrogenase, phosphoglycerate kinase, phosphoglycerate mutase, enolase, and pyruvate kinase. These DEGs, except phosphoglycerate mutase, were downregulated at a 10% DO level, suggesting that low DO can reduce glycolytic flux which may limit pyruvate formation. In addition, the genes encoding pyruvate dehydrogenase, methylmalonate semialdehyde dehydrogenase, hydroxymethylglutaryl CoA synthase (HMGCS), and hydroxymethylglutaryl CoA reductase (HMGCR) were downregulated under 10% DO level. The lower expression of these genes suggests that a low DO level can suppress the conversion of pyruvate to other precursor molecules such as mevalonate and succinyl-CoA.

The genes encoding phosphoglucomutase, 1,4 α-glucan branching enzyme, and glucose-6-phosphate dehydrogenase exhibited lower expression levels under 10% DO. Their lower expression further suggests a reduced glycolytic flux because glucose-6-phosphate (glycolytic intermediate) is one of the substrates in their reactions. The lower gene expression level of 1,4 α-glucan branching enzyme also indicated limited glycogen biosynthesis. Furthermore, the expression of the gene encoding myo-inositol monophosphatase, in the pathway of my-inositol formation from glucose-6-phosphate, was found to be lower at a 10% DO level. The genes encoding triosephosphate isomerase, glycerol-3-phosphate dehydrogenase, and glycerol-3-phosphate O-acyltransferase, involved in glycerol metabolism also exhibited lower expressions at 10% DO level.

Conversely, certain genes involved in the glycerolipid metabolism, including 1-acyl-sn-glycerol-3-phosphate acyltransferase, phosphatidate phosphatase, and diacylglycerol kinase showed higher expression under 10% DO. Similarly, the gene encoding succinyl CoA synthetase, which catalyzes the reversible reaction for substrate-level phosphorylation in the TCA cycle, also exhibited higher expression. The upregulation of this gene possibly suggests a higher flux of succinate to succinyl CoA and ADP formation during growth under a lower oxygen supply. Overall, the above results suggest that a low DO supply induces transcriptional regulation of central carbon metabolism, which may lead to the reduced flux of precursors needed for biomass formation.

**Fig. 3:**
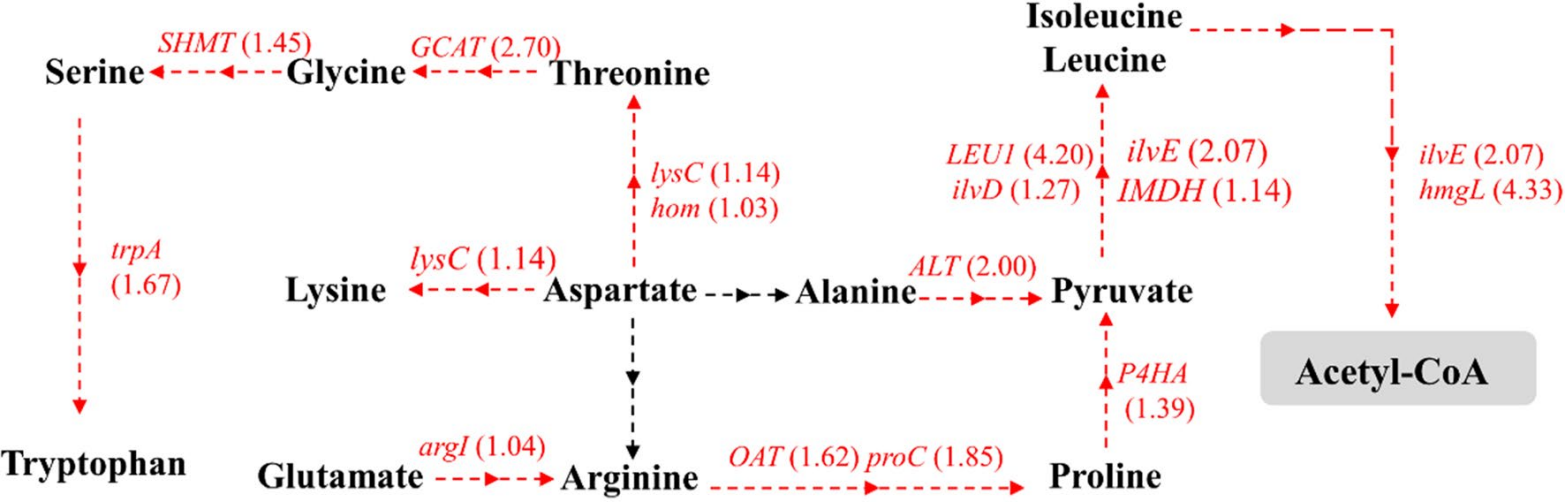
Differential gene expressions in amino acid metabolism between 10 and 30% DO levels at 24 h of fermentation. Genes in red or blue are supported by at least one transcript with significant regulation (|log_2_FC|≥ 1 and FDR < 0.05). Red: upregulated; Blue: downregulated. *trpA* tryptophan synthase alpha chain, *SHMT* glycine hydroxymethyltransferase, *GCAT* glycine C-acetyltransferase, *lysC* aspartate kinase, *hom* homoserine dehydrogenase, *ALT* alanine transaminase, *argl* ornithine carbamoyltransferase, *OAT* ornithine–oxo-acid transaminase, *proC* pyrroline-5-carboxylate reductase, *LEU1* 3-isopropylmalate dehydratase, *ilvD* dihydroxy-acid dehydratase, *ilvE* branched-chain amino acid aminotransferase, *IMDH* 3-isopropylmalate dehydrogenase, *hmgL* hydroxymethylglutaryl-CoA lyase, *P4HA* prolyl 4-hydroxylase

Furthermore, the transcriptomics results revealed upregulation of certain genes involved in amino acid metabolism at 10% DO level (Fig. 3). These genes were found to be particularly involved in the formation of amino acids such as threonine, glycine, serine, tryptophan, alanine, lysine, arginine, proline, leucine, and isoleucine from aspartate. In addition, the *argI* gene encoding ornithine carbamoyltransferase, an enzyme catalyzing the conversion of glutamate to arginine, showed an increased expression. Interestingly, the genes encoding branched-chain amino acid aminotransferase and hydroxymethylglutaryl-CoA lyase, involved in the acetyl-CoA biosynthesis from amino acids, exhibited higher expressions under 10% DO. Taken together, these results suggest that PKU#Mn16 cells mainly derive acetyl-CoA from amino acids when central carbon metabolism gets negatively impacted by low oxygen supply.

### Transcriptional regulation of fatty acid metabolism

**Fig. 4:**
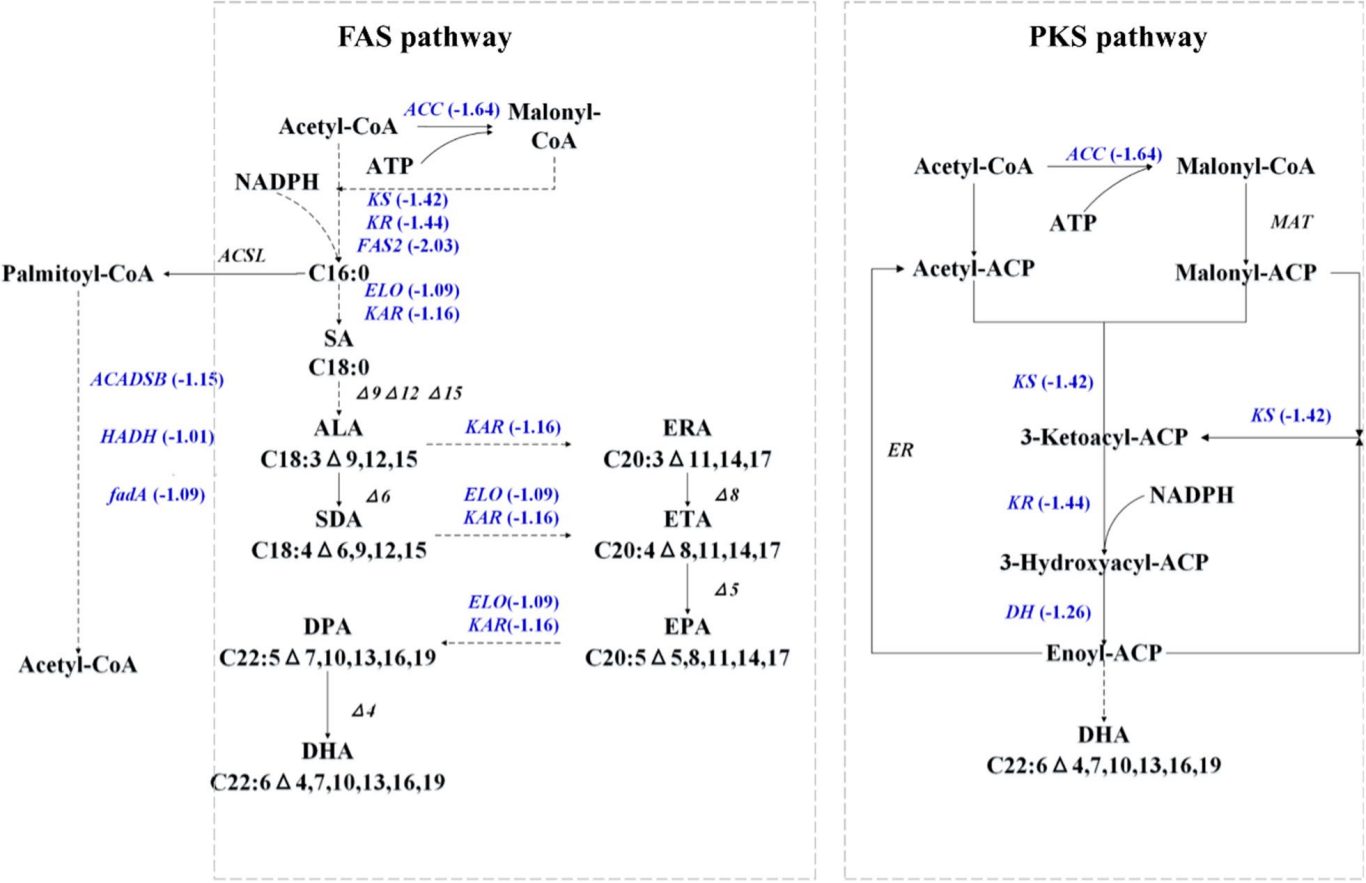
Differential gene expressions in fatty acid metabolism between 10 and 30% DO levels at 24 h of fermentation. Genes in blue are supported by at least one transcript with significant downregulation (|log_2_FC|≥ 1 and FDR < 0.05). *ACC* acetyl-CoA carboxylase, *KS* ketoacyl synthase, *KR* ketoacyl reductase, *FAS2* fatty acid synthase 2, *ELO* very-long-chain 3-ketoacyl-CoA synthase, *KAR* very-long-chain 3-ketoacyl-CoA reductase, *DH* dehydratase, *MAT* malonyl-CoA *ACP* acyltransferase, ER enoyl reductase, *ACADSB* short-chain 2-methyl acyl-CoA dehydrogenase, *HADH* enoyl-CoA hydratase, *fadA* acetyl-CoA acyltransferase, *SA* Stearic acid, *ALA* α-linolenic acid, *SDA* Stearidonic acid, *ERA* Eicosatrienoic acid, *ETA* Eicosatetraenoic acid, *EPA* Eicosapentaenoic acid, *DPA* Docosapentaenoic acid, *DHA* Docosahexaenoic acid

The fermentation results indicated that a 10% DO level can significantly decrease the TFA and DHA accumulation in PKU#Mn16 strain. To further understand the corresponding transcriptional regulation of fatty acid biosynthesis, the expression levels of FAS and PKS pathway genes at 10% DO were compared with those at 30% DO (Fig. 4). The comparative results revealed the downregulation of key genes involved in the FAS and PKS pathways at 10% DO level. These genes included acetyl-CoA carboxylase (ACC), ketoacyl synthase (KS), and ketoacyl reductase (KR). ACC catalyzes the carboxylation of acetyl-CoA to malonyl-CoA and therefore its downregulation suggests that the flux of acetyl-CoA to malonyl-CoA can significantly drop when oxygen supply is low. Similarly, the downregulation of genes encoding KS, KR, fatty acid synthase, and dehydratase indicated that low oxygen supply can negatively affect the formation of long-chain acyl-CoA. Furthermore, very-long-chain 3-ketoacyl-CoA synthase and very-long-chain 3-ketoacyl-CoA reductase were also downregulated, which suggests that a low oxygen supply can reduce the flux of saturated fatty acids into DHA.

In addition to fatty acid biosynthetic genes, the comparative transcriptomics results showed that certain genes involved in fatty acid oxidation were downregulated at a 10% DO level. These DEGs encode short-chain 2-methyl acyl-CoA dehydrogenase, enoyl-CoA hydratase, and acetyl-CoA acyltransferase, which are involved in the β-oxidation of palmitoyl-CoA to acetyl-CoA (Fig. 4). This finding suggests that low oxygen supply can reduce the flux of fatty acids into acetyl-CoA and thereby affect the TCA cycle. Overall, the comparative transcriptomic results indicate that low oxygen supply to PKU#Mn16 cells can significantly affect the transcriptional regulation of fatty acid biosynthesis and oxidation.

### Transcriptional regulation of antioxidative systems

To reveal the effect of low oxygen supply on the antioxidative systems of PKU#Mn16 strain, the expressions of genes involved in enzymatic and non-enzymatic antioxidant defense mechanisms were compared between 10 and 30% DO levels (Table 1). The comparative result showed that genes encoding superoxide dismutase and ascorbate peroxidase in the enzymatic antioxidant system were downregulated at a 10% DO level. Similarly, in the non-enzymatic antioxidant system, the genes encoding HMGCS and HMGCR of the terpenoid biosynthesis pathway, inositol-phosphate phosphatase and galactose dehydrogenase of ascorbate biosynthesis pathway, and carotenoid epsilon hydroxylase of carotenoid biosynthesis pathway were downregulated. These results suggest that low oxygen supply alleviates antioxidative systems through transcriptional regulation.

**Table 1:** Fold change of differentially-expressed genes (|log2FC|≥ 1 and FDR < 0.05) in antioxidative systems

Antioxidants	Pathways	Enzymes	Log_2_FC	Regulation
Enzymatic antioxidants	Peroxisome	superoxide dismutase (SOD)	−1.89	down
		ascorbate peroxidase (APX)	−1.19	down
Non-enzymatic antioxidants	Terpenoid backbone biosynthesis	hydroxymethylglutaryl-CoA synthase (HMGCS)	−2.38	down
		hydroxymethylglutaryl-CoA reductase (HMGCR)	−1.88	down
	Ascorbate biosynthesis	inositol-phosphate phosphatase (VTC4)	−2.52	down
		galactose dehydrogenase (GalDH)	−1.45	down
	Carotenoid biosynthesis	carotenoid epsilon hydroxylase (cyp-13A7)	−1.52	down

RNA sequencing data are provided for 24 h of fermentation. The log2FC represents the comparison between transcriptomics data of experiments conducted under 10% and 30% DO levels

### Intracellular metabolite profiles

**Fig. 5:**
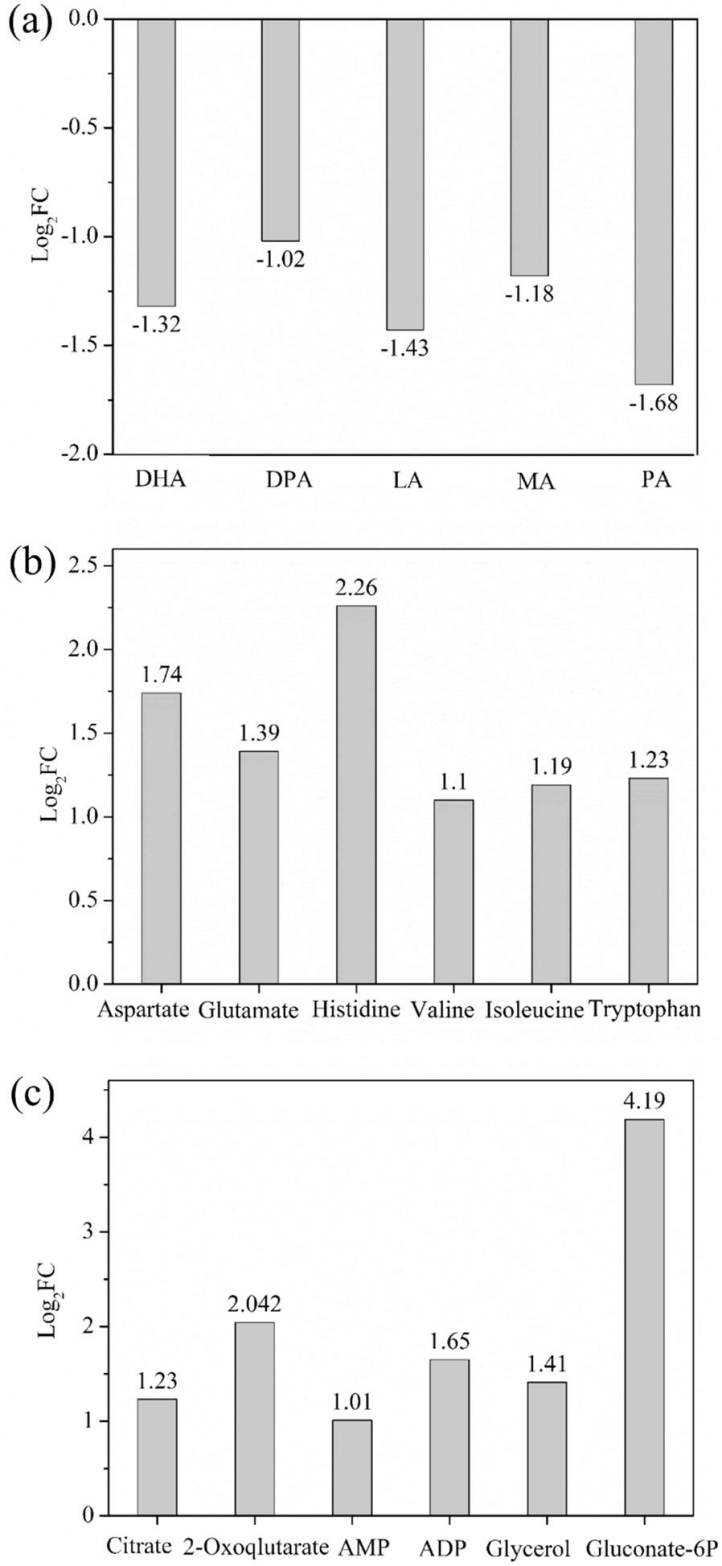
Differential metabolites between 10 and 30% DO levels at 24 h of fermentation. The bar indicates the fold change (log_2_FC) of **a** fatty acids, **b** amino acids, and **c** other metabolites. Metabolites with |log_2_FC|≥ 1 and FDR < 0.05 are shown. *DHA* Docosahexaenoic acid (C22:6), *DPA* docosapentaenoic acid (C22:5), *LA* Dodecanoic acid (C12:0), *MA* Tetradecanoic acid (C14:0), *PA* Hexadecenoic acid (C16:1)

To confirm the effects of DO levels on the metabolism of PKU#Mn16, the intracellular metabolites accumulated under 10% and 30% DO levels were compared using the metabolomics approach. The comparative results revealed several long-chain fatty acids such as DHA, docosapentaenoic acid (DPA), dodecanoic acid (LA), tetradecanoic acid (MA), and hexadecenoic acid (PA) that exhibited lower accumulation at 10% DO level (Fig. 5a). The lower accumulation of these fatty acids supports the downregulation of FAS and PKS pathway genes evident in this study. In addition, certain amino acids like aspartate, glutamate, histidine, valine, isoleucine, and tryptophan showed higher accumulation at 10% DO level (Fig. 5b), which was also correlated with the upregulated genes of the amino acid biosynthesis pathway. Furthermore, certain other metabolites, namely citrate, 2-oxoglutarate, AMP, ADP, glycerol, and gluconate-6-phosphate showed more accumulation at 10% DO level (Fig. 5c). As these metabolites are intermediates of central carbon metabolism, their higher accumulation suggests decreased carbon flow through glycolysis, the TCA cycle, and other pathways of glucose and glycerol metabolism, which generates reducing equivalents and metabolite precursors for biomass growth.

## Discussion

### Impact of dissolved oxygen on DHA fermentation

DO has been reported to play a crucial role in regulating cell growth and lipid accumulation in thraustochytrids [19]. Previous studies suggested that oxygen limitation strategies can result in increased DHA percentage but decreased biomass productivity [13, 26, 27]. In this study, three different DO levels, namely 10%, 30%, and 50% were evaluated on PKU#Mn16 culture to reveal the oxygen-dependent dynamics of cell growth and DHA production. The growth dynamics of the PKU#Mn16 strain observed in our study showed that DO levels can significantly affect the growth rate. A low DO level (10%) can markedly reduce the growth rate in the early stages of fermentation, resulting in less biomass production than 30% and 50% DO levels (Additional file 1: Table S1). However, on prolonged fermentation, until the cells reached their stationary growth phase, a 10% DO level yielded more biomass than that of higher DO levels. A similar pattern of biomass accumulation was reported when *Schizochytrium* sp. S31 was cultivated in fed-batch mode at three different oxygen uptake rates [13]. The higher biomass accumulation at the 10% DO level observed in our study can be explained by the fact that PKU#Mn16 culture consumed more amount of the mixed carbon source (glucose and glycerol) compared with that consumed at higher DO levels (Additional file 1: Fig. S1). Our findings suggest that a 10% DO level is optimal for achieving high biomass accumulation, which could be a result of better consumption of the carbon source by PKU#Mn16 strain in the stationary growth phase.

Previous studies have suggested that stepwise aeration strategies for the cultivation of thraustochytrids can yield the best DHA concentrations compared with that in single-stage cultivation [11, 17]. In this study, the DHA fermentation results indicated that single-stage cultivation of PKU#Mn16 strain with a constant supply of 10% DO level for a period of 144 h can achieve a high amount of DHA. However, with higher DO levels (30% and 50%), the DHA concentrations were relatively lower (Additional file 1: Table S1), which indicates that these high levels of DO saturation provided an oxygen-rich environment that either led to the oxidation of DHA or activated the FAS pathway that is considered to synthesize short-chain fatty acids [28]. Overall, our findings suggest that a constant supply of 10% DO level can be an efficient strategy to improve the overall growth and metabolism of PKU#Mn16 strain and prevent oxidative stress, ultimately resulting in better DHA production.

### Role of dissolved oxygen in carbon and amino acid metabolism

Central carbon metabolism uses a complex series of enzymatic steps to convert sugars into metabolic precursors that ultimately generate the entire cell biomass [29]. A previous study reported that high oxygen supply conditions can significantly upregulate certain genes related to central carbon metabolism, including glycolysis, the pentose phosphate pathway, and the TCA cycle [23]. In this study, the results of comparative transcriptomics revealed transcriptional regulation of central carbon metabolism, suggesting reduced fluxes of metabolic precursors needed for biomass formation at 10% DO level (Fig. 2). This was further supported by the reduced levels of certain precursor metabolites generated in the central carbon metabolism (Fig. 5c). The accumulation of citrate, 2-oxoglutarate, AMP, ADP, glycerol, and gluconate-6-phosphate possibly suggests decreased carbon flow through glycolysis, the TCA cycle, and other pathways of glucose and glycerol metabolism. As these pathways of the central carbon metabolism network generate reducing equivalents and metabolic precursors for biomass formation, their downregulation concurs with the reduced biomass formation in the early stages of fermentation at a 10% DO level.

Apart from the regulation of central carbon metabolism, our study revealed that amino acid metabolism can also get regulated when a 10% DO level is provided to PKU#Mn16 cells. Particularly, the upregulation of ALT, ilvE, and hmgL genes suggests increased conversion of alanine, valine, leucine, and isoleucine to acetyl-CoA. It can thus be speculated that these amino acids can be the main sources of acetyl-CoA at low DO levels. Furthermore, previous studies have reported that some of these amino acids can also play an important role in tolerance and cell viability when microbes combat environmental stress [30]. According to previous studies, tryptophan biosynthetic genes are related to ethanol tolerance of *Saccharomyces cerevisiae* [31]. The increased supplementation of histidine, leucine, and alanine was proposed to improve the stress tolerance of *Saccharomyces cerevisiae* against an excess glucose concentration [32]. When *Schizochytrium* sp. was exposed to an oxygen limitation condition, alanine acted as a protective molecule to cope with the variable conditions due to stress responses [24]. It has been suggested that alanine can be an essential regulatory substance for thraustochytrids to resist the environmental stress of oxygen deficiency. Similarly, glutamate has been reported to play essential roles in cellular metabolism in microorganisms [33]. Glutamine is also found to act as an oxygen stress protectant and can transform into glutamate [24]. In this study, the glutamate accumulation at 10% DO level was significantly increased compared with that at 30% DO level (Fig. 5b). Thus, this result suggests the role of glutamate in stress response against oxygen deficiency during early stages of fermentation. As abiotic stress often leads to the accumulation of amino acids [34, 35], our findings support the fact that certain amino acids can serve as potential stress mitigators of fatty acid biosynthesis in thraustochytrids under low oxygen supply conditions.

### Effects of dissolved oxygen on fatty acid biosynthesis

The analysis of PKU#Mn16 transcriptome revealed several genes encoding fatty acid desaturases involved in the FAS pathway (Fig. 4). However, the critical Δ9, Δ12, Δ15, Δ6, and Δ5 desaturase genes essential for DHA synthesis were not found, suggesting incomplete FAS pathway in this strain. This was in agreement with previous reports [36, 37]. Interestingly, our study found genes related to the PKS pathway, which revealed that the PKS pathway played a more important role in PKU#Mn16 strain. Similarly, the transcriptome profiling of *Aurantiochytrium* sp. PKU#SW7 revealed that DHA biosynthesis occurs via the PKS pathway rather than the FAS pathway [36].

Molecular oxygen is required for fatty acid biosynthesis via the FAS pathway [22]. Whereas the PKS pathway only utilizes the PKS gene cluster to synthesize PUFAs from acyl-CoA de novo [38–40]. A high oxygen supply condition has been shown to upregulate the FAS gene expression in *Schizochytrium* sp. HX-308 [23], suggesting the role of DO in the FAS pathway. In our study, the comparative transcriptomics and metabolomics results indicated that low oxygen supply to PKU#Mn16 cells can significantly affect both fatty acid biosynthesis and oxidation (Fig. 4). ACC, a key enzyme in the fatty acid biosynthesis, produces malonyl-CoA that is used in both FAS and PKS pathways [41, 42]. Our results showed the downregulation of the *ACC* gene, which indicates that malonyl-CoA supply to these pathways may reduce at a 10% DO level, resulting in decreased flux of malonyl-ACP to DHA. The downregulation of KS, KR, and DH genes, and decreased accumulation of several fatty acids, including DHA (Fig. 5a), further support this inference.

Although an optimal oxygen supply condition improves lipid accumulation, a high level of DO can lead to lipid peroxidation [43]. This study found that the fatty acid oxidation pathway was downregulated at the level of 10% DO (Fig. 4), suggesting that a low oxygen supply condition is appropriate for limiting lipid peroxidation. Furthermore, for oleaginous microorganisms such as *Schizochytrium*, a high oxygen supply can result in its conversion into reactive oxygen species (ROS). Interestingly, it was found that some genes involved in antioxidative systems were downregulated at a 10% DO level, which suggests that ROS generation was less at low oxygen supply conditions. A low ROS generation indirectly supports the higher DHA accumulation at the 10% DO level observed in our study.

## Conclusions

Central carbon, amino acid, and fatty acid metabolism are significantly downregulated in the early fermentation stage of *Aurantiochytrium* sp. PKU#Mn16 at low oxygen supply conditions (10% DO). This study revealed that a 10% DO supply to PKU#Mn16 culture can achieve the highest biomass and DHA production at the end of fermentation compared with that achieved at higher DO levels (30% and 50%). However, it was noted that in the early stages of fermentation, the biomass and DHA production could be significantly lower with a 10% DO level than that with higher DO levels. The findings of this study suggest that a constant supply of a low DO level in a variable volume fed-batch fermentation can be an efficient strategy to achieve optimal growth and DHA production.

## Methods

### Microorganism

A previously isolated thraustochytrid [44], *Aurantiochytrium limacinum* strain PKU#Mn16, was used in the present study. The PKU#Mn16 strain was subcultured every two weeks on M4 solid medium (glucose, 20 g/L; peptone, 1.5 g/L; yeast extract, 1 g/L; sea salt, 33 g/L; and agar, 20 g/L). The seed culture was prepared by cultivating a single colony of PKU#Mn16 from the solid M4 medium into flasks (100 mL) containing 30 mL of M4 medium at 28 °C on an orbital shaker at 170 rpm for 24 h. The 24 h grown seed culture (10%, v/v) was then transferred into flasks (1000 mL) containing 300 mL of M4 medium and cultivated at 28 °C on an orbital shaker at 170 rpm for 24 h. The resulting seed culture was used as the inoculum (10%, v/v) for further fermentation experiments.

### Fermentation experiments

Experiments were conducted in a 5 L fermenter (model: SY-9000-V9, Shanghai DongMing Industrial Co. Ltd., Shanghai, China) equipped with DO and pH electrodes, a temperature sensor, an impeller, and an air pump. The working volume of the fermenter was 3 L. The aeration rate was maintained at 300 L/h and three different DO saturation levels, i.e., 10%, 30%, and 50%, were individually studied by altering the agitation speed. A 100% DO saturation level was achieved by setting the agitation at 800 rpm and aeration rate at 500 L/h. The seed culture (300 mL), as described in “Microorganism” section, was inoculated into the fermentation medium (glucose, 30 g/L; glycerol, 30 g/L; yeast extract, 15 g/L; KH_2_PO_4_, 0.25 g/L; sea salt, 33 g/L; and defoamer, 1 mL/L). The fermenter temperature was maintained at 28 ℃ throughout the fermentation. A stock solution (800 g/L) containing equal amounts of glucose and glycerol was prepared and used as a feed to replenish the substrate concentration in the fermenter. About 80–150 g/L of this feed was added to the fermentation broth when the substrate concentration dropped below 25 g/L (Additional file 1: Fig. S1). Samples were taken from the fermenter every 12 h for the analyses of residual carbon source, dry cell weight (DCW), and lipid content. The specific growth rate (µ) of the PKU#Mn16 culture under different DO levels was calculated using Eq. (1) [45].

1 $${B}_{t}={B}_{0}{e}^{\mu t}$$

where B_t_ is the DCW at a given time t, B_0_ is the DCW at t = 0, and µ is the specific growth rate.

### Biochemical analyses

DCW, TFA, and fatty acid composition were determined following methods described in our previous study [46]. The concentrations of glucose and glycerol were determined by enzymatic method using commercial kits (Applygen Technologies Inc., Beijing, China).

### Transcriptome profiling

To investigate the cell response to oxygen saturation, the transcriptional regulation in PKU#Mn16 strain between 10 and 30% saturation levels was analyzed. Three samples (15 mL each) were collected at 24 h of fermentation and analyzed in parallel for each saturation level. The cells were harvested from each sample, frozen with liquid nitrogen, and stored at −80 °C for RNA sequencing. Total RNA was extracted using Trizol reagent (Invitrogen, CA, USA) following the manufacturer's instructions. The RNA fragments were reverse-transcribed to create the cDNA library following the mRNA-Seq Sample Preparation Kit (Illumina, San Diego, USA) protocol. The average insert size for the paired-end libraries was 300 bp (± 50 bp). Total RNA extraction, quality, integrity, and quantity checks were conducted by Lianchuan Bio (Hangzhou, China).

The paired-end sequencing was performed on an Illumina NovaSeq™ 6000 platform (Illumina Inc., USA) at Lianchuan Bio (Hangzhou, China). Cutadapt [47] and in-house Perl scripts were used to remove the reads that contained adaptor contamination, low-quality bases, and undetermined bases. Then, the quality of the clean sequencing reads was verified using the FastQC tool (http://www.bioinformatics.babraham.ac.uk/projects/fastqc/), including the Q20, Q30, and GC content. All downstream analyses were based on high-quality clean data. De novo assembly of the transcriptome was performed with Trinity 2.4.0 [48]. All assembled unigenes were aligned against the NCBI non-redundant (NR) protein database, Gene ontology (GO), SwissProt, Kyoto Encyclopedia of Genes and Genomes (KEGG), Pfam, and eggNOG databases using DIAMOND [49] with a threshold E value < 0.00001. Salmon [50] tool was used to quantify the expression of the transcripts by calculating TPM [51]. The differentially-expressed genes (DEGs) were selected with log2 (fold change) > 1 or log2 (fold change) < −1 and with statistical significance at alpha = 0.05 by R package edgeR [52]. The raw sequencing reads are deposited in NCBI under the BioProject PRJNA901145.

### Metabolome analysis

The changes at the level of intracellular metabolites of PKU#Mn16 strain, in response to different (10% and 30%) DO levels, were evaluated by extracting the metabolites and analyzing them by mass spectrometry. Six samples (total OD_660_ of each sample = 24) were collected at 24 h of fermentation and analyzed in parallel for each DO level. The samples were centrifuged at 10,000 g for 5 min at −4 °C, the supernatant was discarded, and the cell pellets were washed thrice with 20 mL of PBS buffer (pH ~ 7.4). The resulting cell pellets were quickly frozen in liquid nitrogen and then stored at −80 °C until further processing at Lianchuan Bio (Hangzhou, China).

To extract the metabolites, the frozen cell pellets were thawed on ice, and 20 µL of each was quenched with 120 µL of precooled methanol (50% v/v), and then centrifugated at 4000*g* for 20 min. The supernatant was stored at −80 °C before the LC‐MS analysis. All samples were analyzed using a TripleTOF 5600 Plus high-resolution tandem mass spectrometer (SCIEX, Warrington, UK). Chromatographic separation was performed using an ultra-performance liquid chromatography (UPLC) system (SCIEX, UK). An ACQUITY UPLC T3 column (100 mm × 2.1 mm, 1.8 µm, Waters, UK) was used for the reversed-phase separation. The TripleTOF 5600 Plus system was used to detect the metabolites eluted from the column.

The open-access databases, KEGG and HMDB, were used to annotate the metabolites by matching the exact molecular mass data (m/z) to those from the database within a threshold of 10 ppm. Data normalization was performed on all samples using the probabilistic quotient normalization algorithm. The P value was analyzed by Student’s t‐test and adjusted for multiple tests using FDR (Benjamini–Hochberg).

### Statistical analysis

The mean and standard deviation for each measured parameter and the test of significance (Student’s t-test) were computed in Microsoft Excel (Microsoft Corporation, 2016).

## Supplementary Information

**Additional file 1:** **Table S1.** Comparison of the fermentation parameters of Aurantiochytrium sp. PKU#Mn16 under different dissolved oxygen levels. **Table S2.** Annotation summary of de novo assembled unigenes. **Table S3.** Differentially-expressed genes in various metabolic pathways between 10% and 30% oxygen saturation levels. **Figure S1.** Feeding regime for the variable-volume fed-batch culture to evaluate the effects of different oxygen saturation levels (10%, 30%, and 50%) on growth and lipid production of PKU#Mn16 strain. The arrows indicate the point of feeding. **Figure S2.** The profiles of TFA production rates of PKU#Mn16 culture under different oxygen saturation levels. Rate was calculated by dividing the difference in TFA concentration by the change in time between two consecutive data. **Figure S3.** Bar plot of major fatty acids produced by PKU#Mn16 strain under different oxygen saturation levels (10% and 30%). C15, C16, DPA, DHA, and TFA stand for pentadecanoic acid, palmitic acid, docosapentaenoic acid, docosahexaenoic acid, and total fatty acids, respectively. **Figure S4.** Sequence length distribution of transcripts and unigenes

## Data Availability

All data generated or analysed during this study are included in this published article and its supplementary information files.
