# Supplementary material for: Low dissolved oxygen supply functions as a global regulator of the growth and metabolism of Aurantiochytrium sp. PKU#Mn16 in the early stages of docosahexaenoic acid fermentation
Source: Microb Cell Fact. 2023 Mar 15;22:52. doi: 10.1186/s12934-023-02054-w (PMC10015696; doi:10.1186/s12934-023-02054-w)
Supplement: Supplementary file 1 — Additional file 1: Table S1. Comparison of the fermentation parameters of Aurantiochytrium sp. PKU#Mn16 under different dissolved oxygen levels. Table S2. Annotation summary of de novo assembled unigenes. Table S3. Differentially-expressed genes in various metabolic pathways between 10% and 30% oxygen saturation levels. Figure S1. Feeding regime for the variable-volume fed-batch culture to evaluate the effects of different oxygen saturation levels (10%, 30%, and 50%) on growth and lipid production of PKU#Mn16 strain. The arrows indicate the point of feeding. Figure S2. The profiles of TFA production rates of PKU#Mn16 culture under different oxygen saturation levels. Rate was calculated by dividing the difference in TFA concentration by the change in time between two consecutive data. Figure S3. Bar plot of major fatty acids produced by PKU#Mn16 strain under different oxygen saturation levels (10% and 30%). C15, C16, DPA, DHA, and TFA stand for pentadecanoic acid, palmitic acid, docosapentaenoic acid, docosahexaenoic acid, and total fatty acids, respectively. Figure S4. Sequence length distribution of transcripts and unigenes [file 12934_2023_2054_MOESM1_ESM.docx]

Supplementary Information

**Low Dissolved Oxygen Supply Functions as a Global Regulator of Growth and Metabolism of *Aurantiochytrium* sp. PKU#Mn16 in the Early Stages of Docosahexaenoic Acid Fermentation**

Lu Liu^1^, Xingyu Zhu^1^, Huike Ye^1^, Yingying Wen^1^, Biswarup Sen^1*^, Guangyi Wang^1,2,3*^

^1^Center for Marine Environmental Ecology, School of Environmental Science and Engineering, Tianjin University, Tianjin 300072, China

^2^Key Laboratory of Systems Bioengineering (Ministry of Education), Tianjin University, Tianjin 300072, China

^3^Center for Biosafety Research and Strategy, Tianjin University, Tianjin 300072, China

***Corresponding author.**

E-mail: [bsen@tju.edu.cn](mailto:bsen@tju.edu.cn)

gywang@tju.edu.cn

**Table S1** Comparison of the fermentation parameters of *Aurantiochytrium* sp. PKU#Mn16 under different dissolved oxygen levels

Note: Data are the average values of at least triplicate samples with standard deviations (±).

| Time (h) | DO (%) | DCW (g/L) | TFA (g/L) | DHA (g/L) | DHA/DCW (mg/g) |
| --- | --- | --- | --- | --- | --- |
| 12 | 10 | 1.42±0.76 | 0.40±0.27 | 0.06±0.12 | 64.31±39.44 |
|  | 30 | 2.75±0.85 | 0.36±0.05 | 0.10±0.03 | 38.02±17.12 |
|  | 50 | 3.22±0.12 | 0.36±0.13 | 0.11±0.02 | 35.22±6.28 |
| 24 | 10 | 5.30±1.57 | 1.50±0.22 | 0.57±0.13 | 112.50±14.08 |
|  | 30 | 14.22±0.97 | 3.66±0.13 | 1.32±0.07 | 92.78±4.20 |
|  | 50 | 16.27±0.95 | 3.40±0.173 | 1.01±0.04 | 62.37±1.84 |
| 36 | 10 | 13.16±2.35 | 3.37±1.05 | 1.13±0.23 | 90.96±33.05 |
|  | 30 | 23.93±0.76 | 5.27±0.78 | 1.78±0.03 | 74.32±2.68 |
|  | 50 | 26.63±0.34 | 7.62±0.56 | 2.54±0.03 | 95.30±2.28 |
| 48 | 10 | 19.67±2.23 | 5.38±0.77 | 1.80±0.15 | 93.47±17.28 |
|  | 30 | 27.57±1.79 | 6.68±0.39 | 2.43±0.16 | 88.77±11.69 |
|  | 50 | 33.30±2.23 | 9.67±1.78 | 3.59±0.35 | 107.82±6.41 |
| 60 | 10 | 28.33±2.59 | 8.16±1.17 | 2.75±0.39 | 98.91±22.06 |
|  | 30 | 32.97±1.96 | 7.90±0.44 | 2.96±0.11 | 90.35±7.91 |
|  | 50 | 37.60±1.97 | 12.16±0.35 | 4.41±0.22 | 117.25±3.20 |
| 72 | 10 | 35.23±1.53 | 10.33±0.44 | 3.32±0.1 | 94.31±1.61 |
|  | 30 | 37.53±2.09 | 9.68±0.16 | 3.41±0.18 | 90.90±1.89 |
|  | 50 | 37.32±0.95 | 14.02±1.04 | 5.05±0.17 | 135.34±1.40 |
| 84 | 10 | 45.05±3.37 | 13.10±1.35 | 4.41±0.28 | 98.90±13.93 |
|  | 30 | 42.40±2.34 | 10.95±1.55 | 3.96±0.36 | 91.85±9.17 |
|  | 50 | 37.91±0.23 | 14.37±0.26 | 5.11±0.09 | 134.91±3.02 |
| 96 | 10 | 54.10±2.11 | 15.24±0.15 | 5.15±0.23 | 95.42±7.07 |
|  | 30 | 46.12±1.52 | 11.62±0.27 | 4.08±0.01 | 88.48±2.74 |
|  | 50 | 38.21±0.86 | 14.49±1.14 | 5.14±0.38 | 134.45±7.27 |
| 108 | 10 | 55.40±1.45 | 16.26±0.34 | 5.56±0.15 | 100.41±3.43 |
|  | 30 | 46.35±1.72 | 12.29±0.13 | 4.36±0.07 | 94.07±2.20 |
|  | 50 | 37.39±1.29 | 15.02±0.42 | 5.33±0.23 | 142.55±6.48 |
| 120 | 10 | 54.50±3.26 | 17.58±0.55 | 5.70±0.21 | 105.70±8.61 |
|  | 30 | 47.50±2.11 | 13.11±0.56 | 4.73±0.15 | 99.76±6.54 |
|  | 50 | 38.51±0.59 | 15.23±0.66 | 5.30±0.15 | 137.74±4.62 |
| 132 | 10 | 56.00±2.75 | 18.71±0.53 | 6.06±0.19 | 108.69±8.34 |
|  | 30 | 47.05±2.45 | 12.97±1.12 | 4.75±0.23 | 100.91±0.45 |
|  | 50 | 38.07±0.93 | 15.20±0.98 | 5.26±0.14 | 138.41±6.33 |
| 144 | 10 | 56.70±1.84 | 19.02±0.77 | 6.02±0.17 | 106.29±6.05 |
|  | 30 | 46.99±3.19 | 13.00±0.89 | 4.73±0.18 | 100.79±3.14 |
|  | 50 | 38.42±1.07 | 15.10±1.36 | 5.29±0.23 | 137.78±5.00 |

**Table S2** Annotation summary of de novo assembled unigenes.

| Database | Number of unigenes | Ratio (%) |
| --- | --- | --- |
| All | 29097 | 100.00 |
| GO | 8250 | 28.35 |
| KEGG | 5826 | 20.02 |
| Pfam | 8491 | 29.18 |
| swissprot | 7486 | 25.73 |
| eggNOG | 9587 | 32.95 |
| NR | 7497 | 25.77 |

**Table S3** Differentially-expressed genes in various metabolic pathways between 10% and 30% oxygen saturation levels.

| Pathways | Enzymes | Log_2_FC | Regulation |
| --- | --- | --- | --- |
| MAPK signaling pathway | Serine/threonine-protein kinase (CTR1) MAPKKK | 1.99 | up |
|  | Mitogen-activated protein kinase kinase 3 (MKK3) MAPKK | 3.6 | up |
|  | Nucleoside-diphosphate kinase (NME) MAPKK | -2.95 | down |
| Terpenoid backbone biosynthesis | hydroxymethylglutaryl-CoA synthase (HMGCS) | -2.38 | down |
|  | hydroxymethylglutaryl-CoA reductase (HMGCR) | -1.88 | down |
| Oxidative phosphorylation | ATPase | 1.06 | up |
|  | Cytochrome c oxidase assembly protein subunit 17 (COX17) | 2.0 | up |
| Fatty acids metabolism | Long-chain acyl-CoA synthetase (ACSL) | 1.44 | up |
| Glycerophospholipid metabolism | Phosphatidate phosphatase (PLPP1_2_3) | 4.28 | up |
|  | Phosphatidylserine decarboxylase (psd) | 1.42 | up |
| Glycerolipid metabolism | Phosphatidate phosphatase (PLPP1_2_3) | 4.28 | up |
|  | Acyl-CoA dehydrogenase (acd) | -1.58 | down |
|  | Enoyl-CoA hydratase (echA) | -1.01 | down |
|  | Acetyl-CoA acyltransferase (fadI) | -1.03 | down |
| Starch and sucrose metabolism | 1,4-alpha-glucan branching enzyme (glgB) | -4.87 | down |
| Steroid biosynthesis | Delta7-sterol 5-desaturase (ERG3) | 1.29 | up |
|  | Lysosomal acid lipase/cholesteryl ester hydrolase (LIPA) | 1.14 | up |





**Figure S1**. Feeding regime for the variable-volume fed-batch culture to evaluate the effects of different oxygen saturation levels (10%, 30%, and 50%) on growth and lipid production of PKU#Mn16 strain. The arrows indicate the point of feeding.


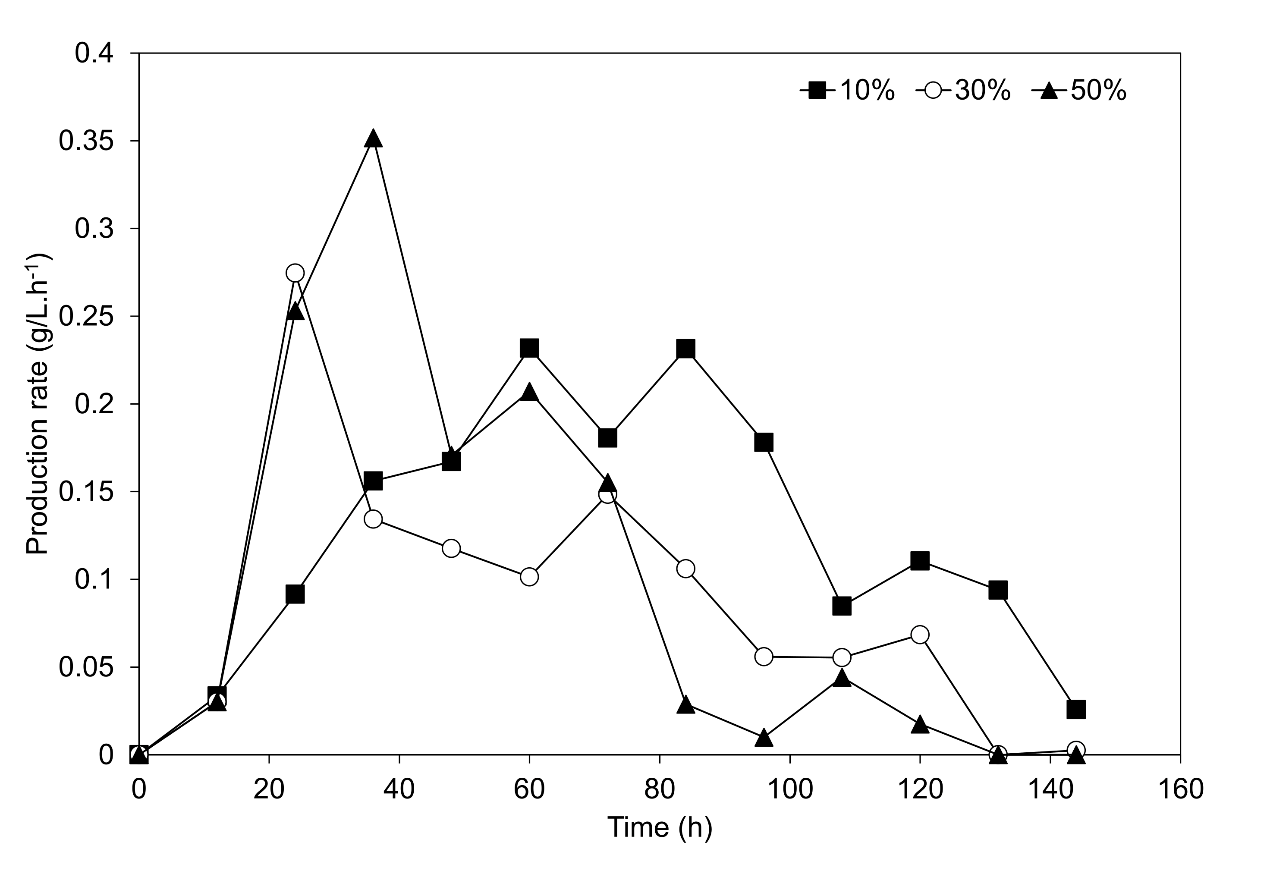


**Figure S2**. The profiles of TFA production rates of PKU#Mn16 culture under different oxygen saturation levels. Rate was calculated by dividing the difference in TFA concentration by the change in time between two consecutive data.





**Figure S3.** Bar plot of major fatty acids produced by PKU#Mn16 strain under different oxygen saturation levels (10% and 30%). C15, C16, DPA, DHA, and TFA stand for pentadecanoic acid, palmitic acid, docosapentaenoic acid, docosahexaenoic acid, and total fatty acids, respectively.


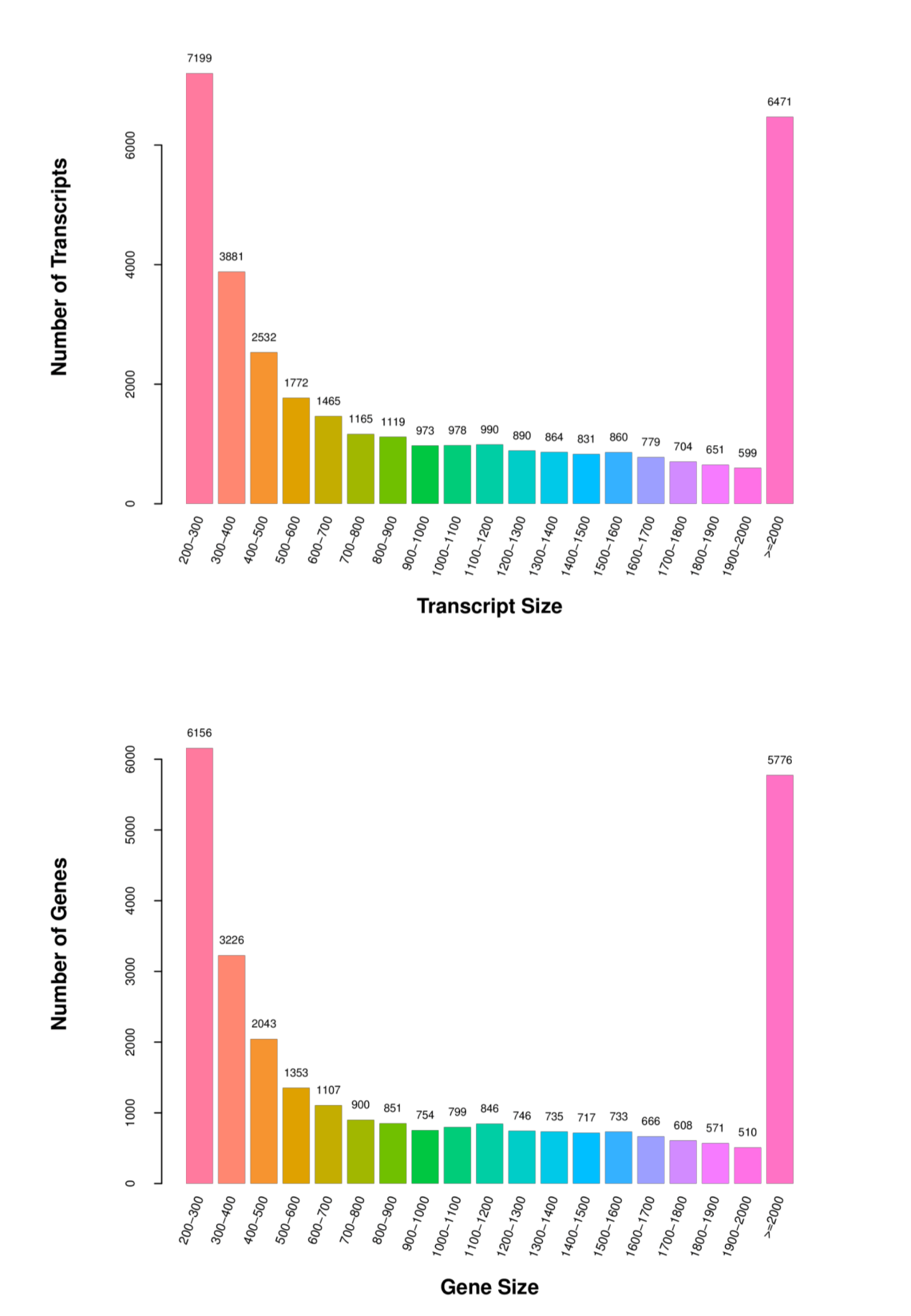


**Figure S4**. Sequence length distribution of transcripts and unigenes.
